# Shocked quartz in distal ejecta from the Ries impact event (Germany) found at ~ 180 km distance, near Bernhardzell, eastern Switzerland

**DOI:** 10.1038/s41598-021-86685-2

**Published:** 2021-04-02

**Authors:** Sanna Holm-Alwmark, Carl Alwmark, Ludovic Ferrière, Matthias M. M. Meier, Sofie Lindström, Gavin G. Kenny, Emma Sheldon, Günter Schweigert, Christoph Spötl, Martin J. Whitehouse, Beda A. Hofmann

**Affiliations:** 1 Niels Bohr Institute, University of Copenhagen, Copenhagen, Denmark; 2 Natural History Museum of Denmark, University of Copenhagen, Copenhagen, Denmark; 3 Department of Geology, Lund University, Sölvegatan 12, 22362 Lund, Sweden; 4 Natural History Museum, Burgring 7, 1010 Vienna, Austria; 5 Institute of Geochemistry and Petrology, ETH Zurich, Clausiusstrasse 25, 8092 Zurich, Switzerland; 6 Naturmuseum St. Gallen, Rorschacher Strasse 263, 9016 St. Gallen, Switzerland; 7 GEUS, Øster Voldgade 10, 1350 Copenhagen, Denmark; 8 Department of Geosciences, Swedish Museum of Natural History, 104 05 Stockholm, Sweden; 9 State Museum of Natural History, Rosenstein 1, 70191 Stuttgart, Germany; 10 Institute of Geology, University of Innsbruck, Innrain 52, 6020 Innsbruck, Austria; 11 Natural History Museum Bern, Bernastrasse 15, 3005 Bern, Switzerland; 12 University of Bern, Institute of Geological Sciences, Baltzerstrasse 1+3, 3012 Bern, Switzerland

**Keywords:** Planetary science, Geochemistry, Mineralogy

## Abstract

Impact ejecta formation and emplacement is of great importance when it comes to understanding the process of impact cratering and consequences of impact events in general. Here we present a multidisciplinary investigation of a distal impact ejecta layer, the Blockhorizont, that occurs near Bernhardzell in eastern Switzerland. We provide unambiguous evidence that this layer is impact-related by confirming the presence of shocked quartz grains exhibiting multiple sets of planar deformation features. Average shock pressures recorded by the quartz grains are ~ 19 GPa for the investigated sample. U–Pb dating of zircon grains from bentonites in close stratigraphic context allows us to constrain the depositional age of the Blockhorizont to ~ 14.8 Ma. This age, in combination with geochemical and paleontological analysis of ejecta particles, is consistent with deposition of this material as distal impact ejecta from the Ries impact structure, located ~ 180 km away, in Germany. Our observations are important for constraining models of impact ejecta emplacement as ballistically and non-ballistically transported fragments, derived from vastly different depths in the pre-impact target, occur together within the ejecta layer. These observations make the Ries ejecta one of the most completely preserved ejecta deposit on Earth for an impact structure of that size.

## Introduction

Impact cratering is a ubiquitous process throughout the solar system, although its effects are obscured and/or removed on Earth due to its active geological nature (e.g., Ref.^1^). During the formation of an impact crater, fractured, crushed, melted, and vaporized rocks and minerals are excavated from the opening transient cavity, forming an ejecta layer that thins away with increasing distance from the crater, e.g., Refs.^2,3^ and references therein. The distribution of ejecta around an impact crater varies, and is dependent on various factors, including impact angle, impact velocity, volatile content, cohesiveness, and composition of target rocks, as well as the size of the impactor, e.g., Ref.^4^. Ejecta layers known on Earth are described as either proximal or distal^2,3^. The division between the two is either made on the basis that proximal ejecta occurs at less than 5 crater radii from the center of the crater, and ejecta outside of that limit is then considered to be distal, e.g., Ref.^3^, or distal ejecta are defined as ejecta occurring outside of the continuous ejecta blanket, which extends 2–3 crater radii from the center of the crater^5^.

Few distal impact ejecta layers have been identified on Earth^6^, partly because they are rarely preserved, but also because of the difficulties in identifying these layers in the rock record. Only a handful of ejecta layers have been tied to their respective source crater^2,6^. This renders their formation relatively poorly understood, in particular the mode of emplacement and the provenance of ejecta components. Characterized distal ejecta layers can give important clues on impact events for which the impact crater is not preserved (or unknown), as for the Australasian tektite strewn field, e.g., Refs.^2,7,8^, or act as the only source of information on the Earth’s early bombardment history^2^.

Impact ejecta can be identified and characterized based on, e.g., the presence of shock metamorphic features. In fact, the occurrence of shocked quartz in Cretaceous–Paleogene (K–Pg) boundary layers around the world was key evidence for understanding that ejected material from the Chicxulub impact had spread across Earth^9^. Shocked quartz is characterized by the presence of planar deformation features (PDFs)^1^. They consist of thin, straight, planar lamellae of amorphous or high-dislocation-density material, forming sets of features spaced 2–10 µm apart, oriented along rational crystallographic orientations, e.g., Refs.^1,10^. There is only one diagnostic macroscopic indicator for shock metamorphism, namely shatter cones. They are macro- to mesoscopic pervasive, curved to curvilinear fractures decorated with more or less divergent striations that radiate from an apex or an apical area, e.g., Ref.^1^ and references therein.

The aim of this study is to confirm and refine the origin, mechanism of emplacement, and age of the enigmatic “Blockhorizont”, known from three different localities in eastern Switzerland, e.g., Ref.^11^, which has been previously proposed to be distal impact ejecta from the Ries impact structure (Germany)^12,13^. An important aspect motivating this work is that failing to simulate important components of the ejecta layer at the Ries impact structure is explained by a lack of knowledge of some physical processes that act when impact ejecta is formed^14^. Studies of impact ejecta deposits, such as the one presented here, are thus crucial for the possibility to further constrain these models.

We apply a multi-disciplinary approach that involves detailed petrographic analyzes, Universal stage (U-stage) characterization of shocked quartz grains (for confirmation of the mode of formation of the planar microstructures and for the estimation of the experienced shock pressures), U–Pb dating of bentonite horizons adjacent to the Blockhorizont to determine the age of deposition, as well as whole-rock element analysis, C- and O-isotopes, and paleontology to constrain the provenance of the ejecta components.

## Previous works on the Blockhorizont and the Ries impact structure

**Figure 1:**
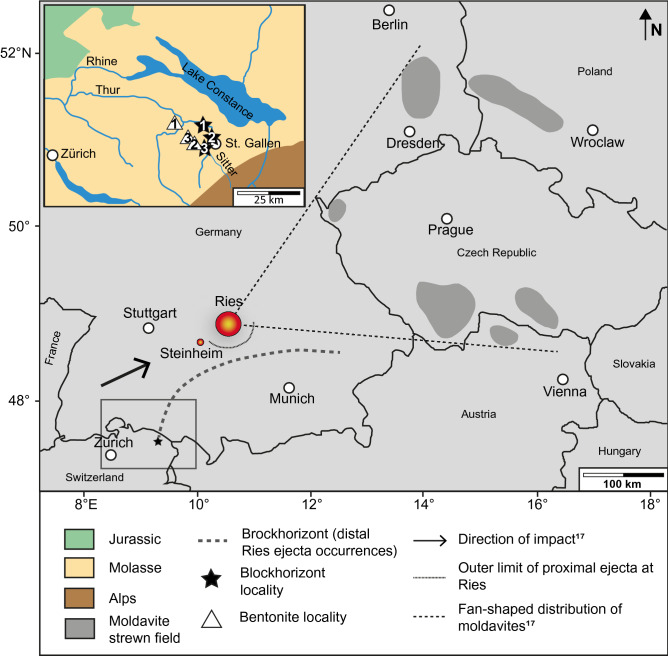
Map showing the location of the Ries and Steinheim impact structures, along with the distribution of proximal and distal impact ejecta from the Ries. Inset is a close-up showing the general geology of the area with the three localities where the Blockhorizont has been recognized in Switzerland (stars), and sample localities for the bentonite samples (triangles). Gray dashed line connects localities of the Brockhorizont, where limestone clasts have been found and are interpreted as distal Ries ejecta (note that this does not mean that the distal ejecta from Ries do not occur beyond this line; e.g. Ref.^16^), with the Blockhorizont in Switzerland. Note the historical difference between the names “Blockhorizont” (the name for the enigmatic horizon in Switzerland^11^) and “Brockhorizont” (the name of the horizon located in molasse in southern Germany containing distal ejecta particles also interpreted to come from the Ries). Star 1: Bernhardzell (samples investigated in this study), star 2: Erlenholz, and star 3: Tiefenbachtobel. Triangle 1: Bischofszell bentonite (investigated in this study), triangle 2: Tiefenbachtobel bentonite (investigated in this study), and triangle 3: Mollen-Waldkirch bentonite. Large map modified from Ref.^17^, and small, inset map in top left corner modified from Ref.^15^.

**Figure 2:**
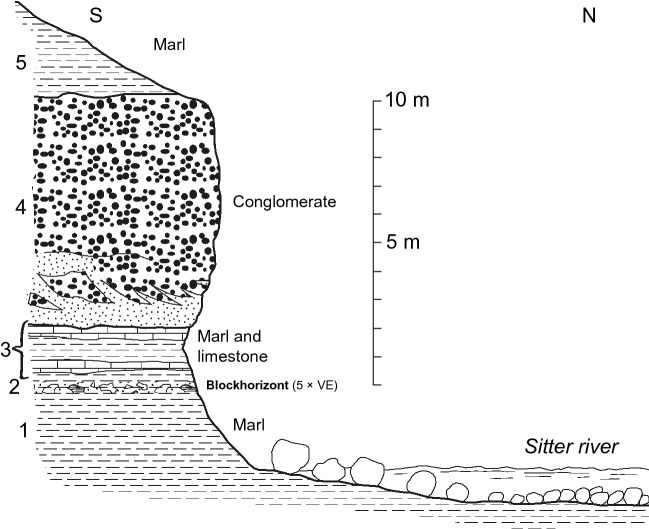
Stratigraphic section of the Bernhardzell Blockhorizont site (this study). (1) Marl, (2) Blockhorizont, (3) Marl and marl with thin layers of limestone, (4) Conglomerate of the Hörnli alluvial fan, and (5) Marl. All units shown here belong to the Upper Freshwater Molasse of the North Alpine Foreland Basin. Thickness of Blockhorizont exaggerated (×5) for visibility. After Ref.^18^.

**Figure 3:**
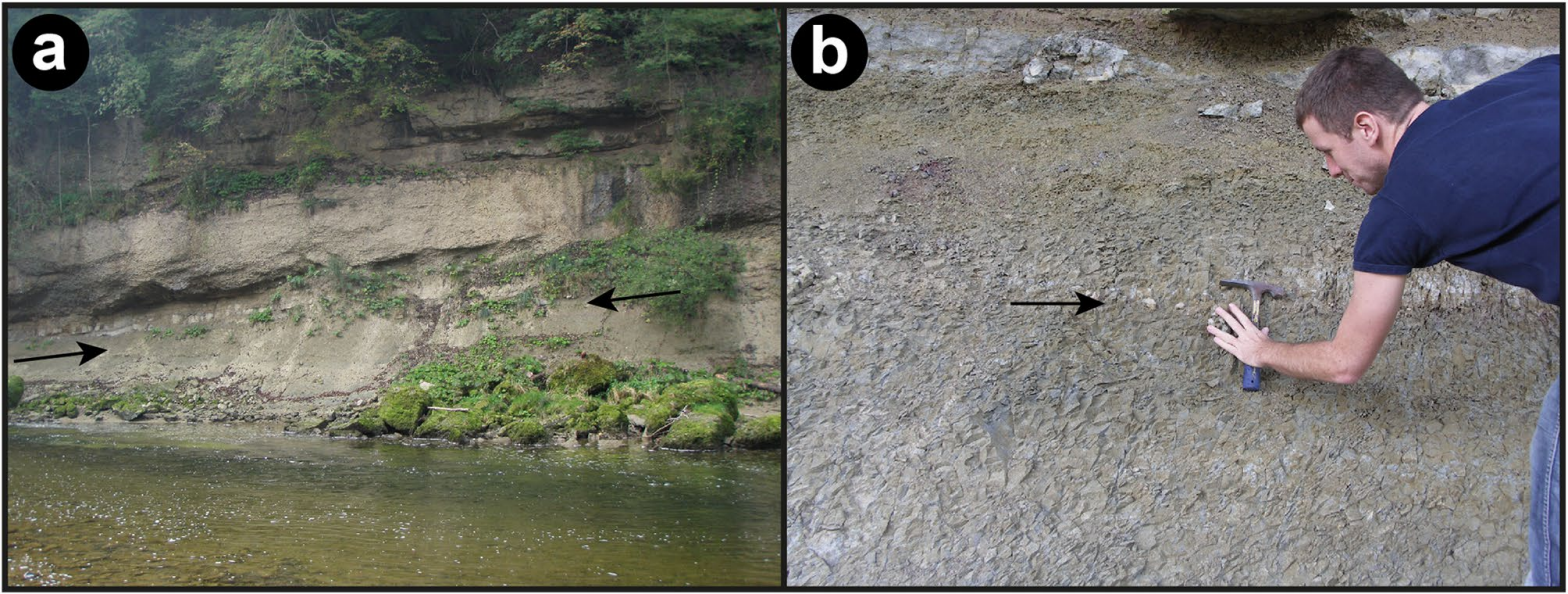
Photographs from the Blockhorizont sampling site near Bernhardzell. (**a**) depicts the Molasse section containing the Blockhorizont (marked with arrows). (**b**) Close-up of the section, with the Blockhorizont marked with an arrow (photograph by Rainer Wieler).

The Blockhorizont is a ~ 10 cm thick layer of marl containing randomly distributed angular blocks and smaller fragments of rocks, mainly limestone and red mudstone, and mineral grains. The layer is not defined by sharp boundaries in the field, but is clearly visible due to the presence of fragments of various sizes. Individual mineral grains and rock fragments in the layer vary between < 1 mm to 40 cm in size. The horizon is known from three different localities in north-eastern Switzerland, embedded in Middle Miocene Upper Freshwater Molasse, in the North Alpine Foreland Basin^11,12,15^ (Figs. 1, 2, 3). The depositional environment for the horizon is on a large alluvial fan, and the adjacent strata is typical for the Miocene freshwater Molasse, i.e., dominated by very fine-grained marls with minor conglomerates, sandstones, and limestone^15^. The Blockhorizont was initially proposed to be impact-derived^15^ based on the presence of shatter cone fragments, and Ref.^12^ suggested the Ries impact structure (~ 180 km away) as its possible source. These authors also reported the detection of suspected shocked quartz within the layer but did not provide any detailed descriptions or crystallographic constraints to confirm this.

The 26 km diameter^13^ Ries impact structure (southern Germany; Fig. 1) is one of the best-preserved complex impact structures on Earth, e.g., Ref.^13^. The structure formed at 14.808 ± 0.038 Ma^17^. The nearby ~ 3.8 km in diameter Steinheim impact structure^19^ is considered to have formed simultaneously, although this has been challenged in a recent publication^20^. The target bedrock is composed of sedimentary rocks of Triassic to Cenozoic age underlain by crystalline basement (Ref.^21^ and references therein; Supplementary Fig. S1), and all these types of target rocks occur as clasts in the proximal impactites at the Ries^13^.

The Ries has one of the best exposed and preserved ejecta deposits documented on Earth to date, which includes a continuous, double-layer, proximal ejecta blanket where the so-called Bunte Breccia, a polymict breccia with block sizes < 25 m, occurs together with megablocks with sizes > 25 m^13^. The Bunte Breccia is overlain by the more patchy, melt-bearing impact breccia, called Ries suevite^21^. In addition, the proximal impactites also comprise impact melt rock^22^. The continuous ejecta blanket reaches a maximum radial extent of 44 km^23^. The Ries ejecta deposits also include distal impact ejecta (Fig. 1) forming the Brockhorizont^13,15^ and references therein, that occur in the Molasse of the North Alpine Foreland Basin, and that contains large (up to meter-sized) isolated Upper Jurassic limestone blocks which are sometimes shatter cone-bearing (e.g., Refs.^13,24,25^ and references therein), as well as the Moldavite tektite strewn field, e.g., Refs.^13,26^ and references therein, that extends for ~ 200–500 km from the center of the Ries to the east-northeast (Fig. 1). The location of the Moldavite tektite strewn field with respect to the locations of the Ries and Steinheim impact structures suggests a direction of impact from the west-southwest^19^ (Fig. 1).

## Bentonites in the Swiss Molasse Basin

A number of bentonite horizons occur in the Swiss Molasse Basin, ranging in age from 14.2 to 15.3 Ma^27^. The occurrence of a bentonite horizon at Tiefenbachtobel (in a continuous profile), located just 875 m northwest of one of the exposures of the Blockhorizont in Switzerland (Fig. 1), was described by Ref.^15^. Stratigraphically, the bentonite layer is 70 m above the impact horizon^15^. Note that there are currently, as far as we know, no exposures of the Blockhorizont and a bentonite at the exact same locality.

The bentonite at Tiefenbachtobel can be correlated with a bentonite from Bischofszell, for which consistent age determinations of 14.28 ± 0.24^28^, and 14.417 ± 0.023 Ma^27^ are available (see also our results below), based on lithostratigraphy. The sediments of the Miocene Molasse in this area are close to horizontal and some thick conglomerate beds can be correlated from one outcrop to another (e.g., Ref.^29^). There is another outcrop of bentonite at Mollen-Waldkirch, just 4.2 km NE of Tiefenbachtobel. The Mollen bentonite is equivalent in age to the Bischofszell bentonite^28^ and can be correlated with the one at Tiefenbachtobel based on lithostratigraphy. While the correlation between bentonites occurring in eastern Switzerland (Winterthur, Mollen-Waldkirch, Rengishalden-Bischofszell, and Schosstobel) was uncertain^30^, recent zircon ages obtained by the British Geological Survey have now shown that all these horizons correspond to the same horizon^28^, which is also supported by lithostratigraphy^29^. The weighted mean age, as calculated by us, of the four localities is 14.34 ± 0.03 Ma.

## Results and discussion

Analysis of rock fragments (mudstone, limestone, and crystalline bedrock fragments were observed by us) and sand grains from three individual samples of the Blockhorizont from the Bernhardzell locality (Fig. 1) in eastern Switzerland (Table 1) shows that, compared to samples from 20 to 70 cm above and below, the Blockhorizont is significantly enriched in both rock particles > 1.5 mm, including limestone fragments, and HCl insoluble particles > 0.3 mm. There are no rock fragments > 1.5 mm in the investigated samples from above and below the horizon, whereas the Blockhorizont contains 1.8–2.5% of such larger fragments. The quantity of acid insoluble smaller particles is on the order of ~ 350–800 ppm in the Blockhorizont and ~ 20–60 ppm above and below the horizon.

**Table 1:** Concentration of rock/mineral grains in marls from the Bernhardzell sampling locality (three independently analyzed Blockhorizont samples).

Position^a^ (cm)	> 1.5 mm total (%)^b^	HCl insoluble > 0.3 mm (ppm)^c^
40	0	54
20	0	23
0	1.8, 2.1, 2.5	763, 353, 814
− 20	0	63
− 70	0	60

^a^Relative to center of visual ejecta layer with limestone fragments.

^b^Includes limestone fragments.

^c^Concentration (ppm = mg/kg) of HCl insoluble silicate grains > 0.3 mm in diameter in the bulk sample.

**Figure 4:**
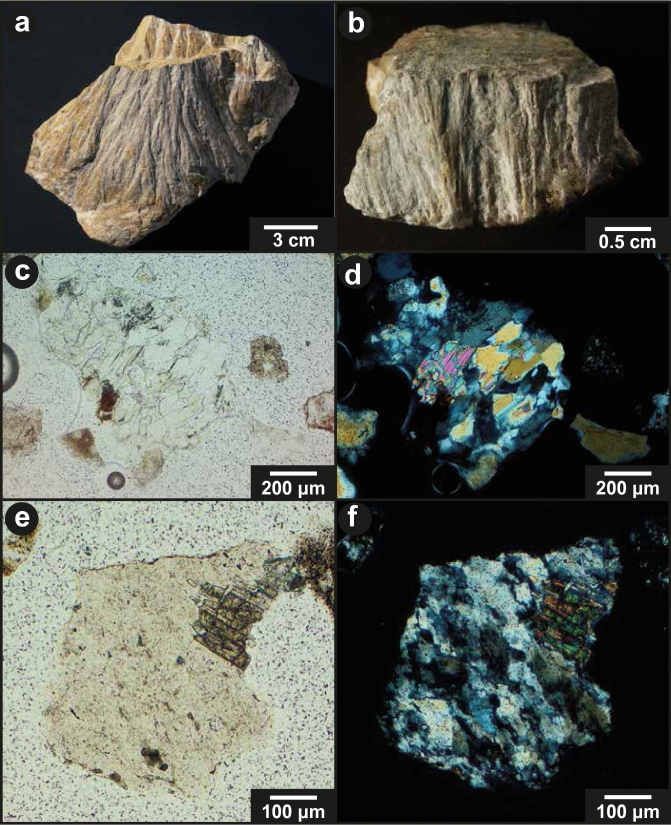
Clasts from the Blockhorizont at Bernhardzell (i.e., our sampling locality of the Blockhorizont). (**a**,**b**) Limestone fragments with shatter cones (Natural History Museum Bern collection; Photographs taken by Peter Vollenweider, Natural History Museum Bern). (**c**) Rock fragment with quartz, muscovite, and a mafic phase (plane-polarized light; PPL). (**d**) Fragment from (**c**), in cross-polarized light (XPL). (**e**) Rock fragment with quartz and titanite (PPL). (**f**) Fragment from (**e**), in XPL.

Rock fragments were further characterized in the investigated thin sections (Fig. 4; Supplementary Fig. S2). In the thin sections, we observed lithic clasts up to 1 mm in size. The clasts consist of quartz and mica (biotite and muscovite) alongside other strongly altered minerals. The clasts are typically angular in shape, and individual mineral grains in those clasts are not rounded, with one possible exception (Supplementary Fig. S2a,b). We have observed PDFs in quartz grains in these angular lithic clasts (Supplementary Fig. S2c–e).

### Characterization of the quartz grains

**Figure 5:**
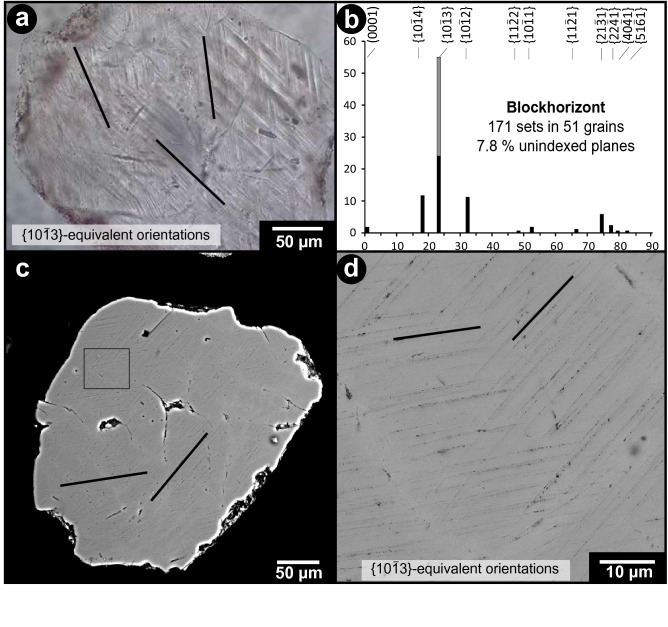
(**a**) Microphotograph of a quartz grain from the Blockhorizont displaying three PDF sets. (**b**) Histogram of the absolute frequency percent of indexed PDFs in quartz from the Blockhorizont. PDFs that plot in the overlapping zone between the {${10 \overline{1} 4}$} and {${10 \overline{1} 3}$} orientations are plotted in grey on top of the uniquely indexed {${10 \overline{1} 3}$} planes (as recommended by Ref.^31^). (**c**) Backscattered electron image of a shocked quartz grain from the Blockhorizont. (**d**) Close-up of PDFs from the grain in (**c**) showing the decorated nature of the lamellae.

We have conducted the first detailed investigation of quartz grains from the Blockhorizont. The grains range from 140 to 630 μm in size, are mainly monocrystalline, and constitute individual mineral grains. Shock metamorphic features in the form of PDFs were detected in ~ 5% of the investigated quartz grains (Fig. 5). The orientations of PDFs were measured in 51 quartz grains from the Blockhorizont, resulting in a total of 171 measured sets (Fig. 5b; for detailed results see Supplementary Table S1). The PDFs appear largely undecorated at the optical microscope scale, but decorations are visible when the grains are studied with the scanning electron microscope (SEM; Fig. 5c,d). The number of PDF sets per quartz grain vary from one to seven, with an average of 3.4 sets per grain.

Based on our investigations of the PDF orientations we can estimate that the quartz grains experienced shock pressures of up to ~ 25 GPa, based on the occurrence of grains with sets parallel to the {${10 \overline{1} 2}$}-orientation (i.e., 27% of the quartz grains have PDFs parallel to this specific orientation), sometimes multiple such sets in a grain, and without the presence of basal (0001) PDFs (see discussion in Ref.^32^ and references therein). The average shock pressure for the investigated quartz grains in the sample was estimated after assigning each grain with a specific pressure tied to the PDF population of that specific grain. This gave an average shock pressure of ~ 19 GPa (see details of the method in Ref.^32^). No shock metamorphic features were detected in the investigated mudstone samples.

The shocked quartz grains display typical signs of being affected by abrasion (Fig. 5a,c). The effect of this abrasion, however, is somewhat limited as the average angularity of these grains according to the Pettijohn scale is 2, i.e., angular.

### Geochemical analyses

**Figure 6:**
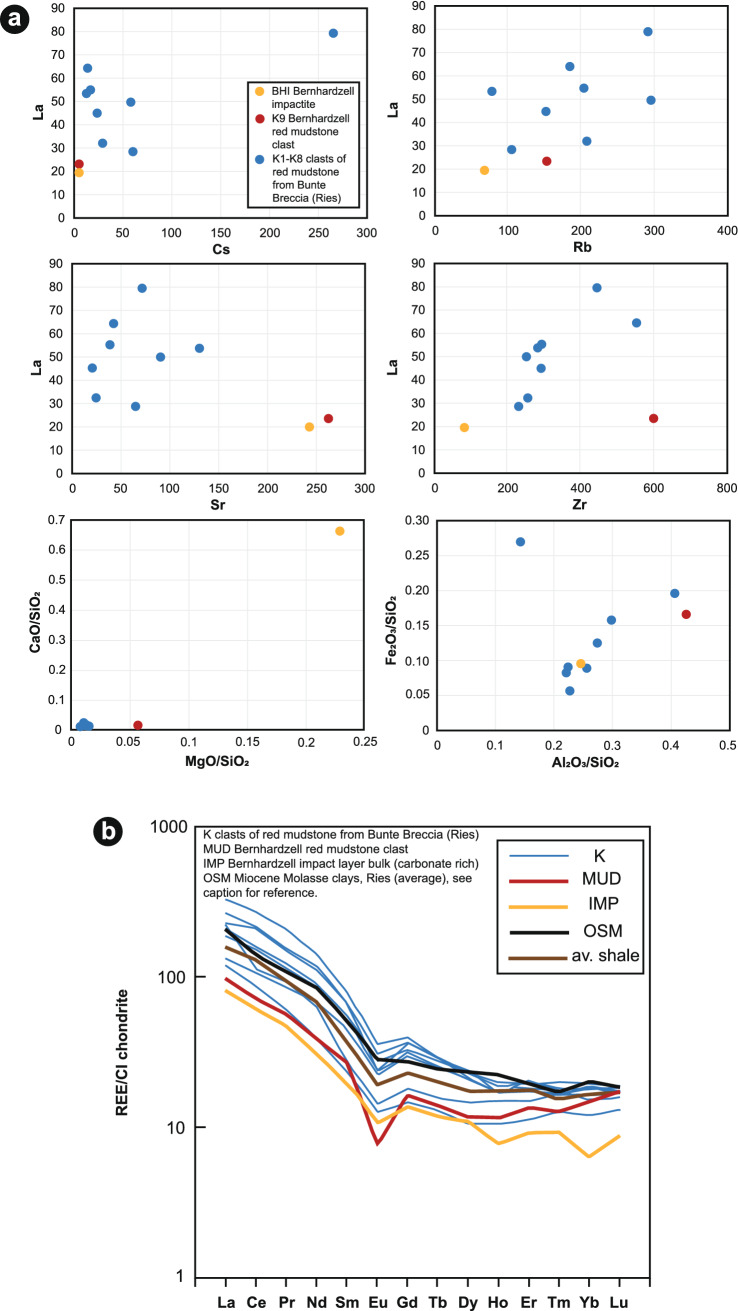
(**a**) Whole-rock geochemical plots showing the variations in La, Cs, Rb, Sr, Zr, CaO, SiO_2_, MgO, Fe_2_O_3_, and Al_2_O_3_ for the Bernhardzell samples compared with samples from the Ries. (**b**) REE plot of Blockhorizont samples compared with samples from the Ries. Data for Miocene Molasse clays from Ries were obtained from Ref.^65^.

Compared with Keuper clay from the Ries structure (clasts from the Bunte Breccia), the red mudstone occurring as fragments in the Blockhorizont shows some differences in major and trace element concentrations, especially for Si/Al, Cs, and REEs, as well as in mineralogy, implying that the mudstone from the Blockhorizont is not of Keuper origin (Fig. 6; for full results, see Supplementary Tables S2 and S3). The low Cs concentration (~ 5 ppm) of the mudstone found as a clast within the Blockhorizont (Fig. 6a) clearly distinguishes it from the Ries Keuper clay samples, which in our analyzes are shown to have Cs concentrations between 14 and 266 ppm ($\overline{\mathrm{x}}$ 60 ppm). Triassic rocks of the studied region are typically enriched in Cs^33^.

Three aliquots of each of the three limestone clasts (two of them with shatter cones) from the Blockhorizont yielded δ^13^C and δ^18^O values between 2.0 and 2.5‰, and between − 2.1 and − 2.4‰, respectively (Supplementary Fig. S3, Supplementary Table S4). These are typical values for Upper Jurassic marine limestones from north of the western Tethys^34^. In addition, three samples of a shatter cone from the Steinheim structure show δ^13^C values between 2.1 and 2.2‰, perfectly matching the values of the Blockhorizont clasts. The δ^18^O value of this shatter cone sample (− 3.9‰) is slightly more negative than the values of the limestone clasts from the Blockhorizont. Δ^18^O values of carbonate rocks are commonly more variable than δ^13^C values, because the oxygen isotopic composition is more prone to diagenetic alteration (e.g., Ref.^35^). The small difference in δ^18^O between clasts of the Blockhorizont and the shatter cone sample from Steinheim therefore is still consistent with the hypothesis that the limestone clasts are derived from Upper Jurassic carbonate units in the Ries/Steinheim area, for which^36^ reported δ^18^O values ranging from − 2.6 to − 3.8‰.

### Palynology and paleontology

The red mudstone from the Blockhorizont contains palynomorphs that are either of late Jurassic or Miocene age, or long-ranging taxa (see Supplementary Table S5 and Supplementary Plate 1 for full results of the palynological investigation). The small angiosperm pollen and *Taxodiumpollenites hiatus* probably represents the flora growing at the time of impact and could have been incorporated into the sample at or immediately after deposition at Bernhardzell. This may also be the case for two dinoflagellate specimens (Supplementary Plate 1: A,B and H), which resemble the taxa *Pseudokomewuia granulata* and *Cleistosphaeridium placacanthum*, respectively, which are both known from Miocene strata of NW Europe (see e.g., Ref.^37^). According to Ref.^38^, red claystones are typical Miocene deposits of the Upper Freshwater Molasse in the Ries area, and these deposits pre-date the formation of the impact structure.

The remaining spores and pollen, the dinoflagellate cysts, acritarchs, and prasinophytes may be derived from marine Late Jurassic strata that was present in the pre-impact stratigraphic sequence were the Ries crater was excavated, e.g., Ref.^13^. The partial dinoflagellate cyst in Supplementary Plate 1, C,D, shows some resemblance to the late Tithonian to early Berriasian *Gochteodinia virgula*, but also to members of the Jurassic-Cretaceous genus *Prolixosphaeridium*^39^.

The limestone clasts we have investigated are derived from Upper Jurassic strata, as determined based on ammonites occurring in some of these clasts (Supplementary Fig. S4). The ammonites are likely from the “Untere-Felsenkalke Formation” of the Swabian facies which corresponds to an early Late Kimmeridgian age. No nannofossils were detected in our analysis.

### Age of the bentonite horizons

**Figure 7:**
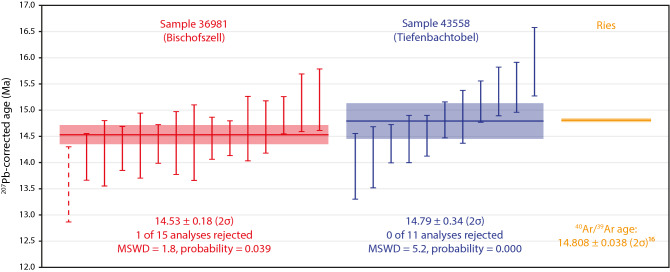
Comparison of results from U–Pb dating of two bentonites (this study) with published ^40^Ar/^39^Ar age^17^ for the Ries impact structure. Weighted mean ages were calculated using the Isoplot 4.15 software^40^. *MSWD* mean square of weighted deviates.

For this study, the bentonite horizon at Tiefenbachtobel (which has not previously been dated) as well as one occurring at Bischofszell (Fig. 1), were sampled with the aim to constrain the age of the Blockhorizont. Results of U–Pb dating of zircon grains from the two bentonite horizons are presented in Fig. 7, Supplementary Fig. S5, and Supplementary Table S6 (see also an extended discussion in the Supplementary Information). Sample from the Bischofszell bentonite (NMBE 36981) gave a weighted mean ^207^Pb-corrected age of 14.53 ± 0.18 Ma (2σ, mean square of weighted deviates [MSWD] = 1.8, probability = 0.039, one of 15 analyses excluded). The new age is consistent with the 14.28 ± 0.24 Ma age obtained from this bentonite by Ref.^28^, and the 14.417 ± 0.023 Ma age obtained from the same bentonite by Ref.^27^. We thus confirm the age of the Bischofszell bentonite.

Sample from the Tiefenbachtobel bentonite (NMBE 43558) displays more spread in U–Pb dates. Eleven Miocene-aged grains give a weighted mean ^207^Pb-corrected age of 14.79 ± 0.34 Ma (2σ, MSWD = 5.2, probability = 0.000, none of 11 analyses excluded), indistinguishable from the age of sample NMBE 36981. The new U–Pb data are fully consistent with the Tiefenbachtobel bentonite having the same depositional age as the Bischofszell bentonite, despite not conclusively proving it.

### Age of the Blockhorizont

The absolute age of the Blockhorizont can be estimated from the stratigraphic position of the related dated bentonite horizons. The bentonite layer at Tiefenbachtobel is located 70 m above the impact horizon^15^ and this 70 m sequence of strata is typical of the whole Miocene molasse sequence in the area (see above and also e.g., Ref.^29^). Therefore, we can assume a mean sedimentation rate of 150 to 200 m/Myr for the Miocene Upper Freshwater Molasse (based on Ref.^30^), making the Blockhorizont 0.35–0.47 Ma older than the bentonite in the Tiefenbachtobel profile.

Our age determination of the Bischofszell bentonite (14.53 ± 0.18 Ma; Fig. 7) is consistent with ages presented previously^27,28^. Considering the results of our analysis, we conclude that Tiefenbachtobel has the same age as Bischofszell, and therefore we use the most precise available determination of 14.417 ± 0.023 Ma for the Bischofszell bentonite^27^ as the age of the Tiefenbachtobel bentonite. Therefore, together with the age difference between the bentonite and the impact layer of 0.35–0.47 Ma, an age of 14.74–14.91 Ma can be estimated for the Blockhorizont impact ejecta layer. This age overlaps the age estimated for the deposition of the Brockhorizont, ~ 14.9–15.1 Ma, which was achieved using a bracketing method^27^, see also discussion in Refs.^41,42^, and indistinguishable within error from the age of the Ries impact of 14.808 ± 0.038 Ma^17^. Additionally, the palynological results also support a Miocene age (pre-late Tortonian) for the Blockhorizont (Supplementary Table S5).

### Is the Blockhorizont impact ejecta from the Ries impact?

The present study with the characterization of shocked quartz grains unequivocally confirms that the Blockhorizont was formed by impact, supporting previous works based on the finding of shatter cone clasts^12^. The angularity of the quartz grains (average = 2, i.e., angular; Fig. 5) indicates only modest reworking (see e.g., Refs.^43,44^). Hence, long distance transport of the material by fluvial processes from the Ries to the outcrop locality is highly unlikely (compare fluvially transported grains from the Ries in Ref.^45^). However, our main argument against fluvial transport from Ries is the fact that all the known Blockhorizont localities are situated on an alluvial fan system consisting of material transported from the Alps, thus from the south (see Ref.^30^). In addition, it is very unlikely that the concentration of shocked quartz grains would be as high as observed in this study if the grains would have been fluvially transported for ~ 200 km. Fluvial transportation is expected to dilute the population of shocked quartz grains, especially over ~ 200 km distance. This was demonstrated e.g., in the case of investigation of detrital shocked quartz grains derived from the Vredefort impact structure and fluvially transported^46^. It is also difficult to imagine how large limestone clasts, up to 40 cm in size, would be fluvially transported for such a large distance, especially since both fragments from the Blockhorizont, as well as those from the Brockhorizont^16^, are angular in shape. Several models^14,19,47^ show that large rock fragments from the Ries ejecta, as large as the ones described from the Blockhorizont, could have been ballistically transported as far as the location investigated here.

Our age estimate of 14.74–14.91 Ma for the Blockhorizont impact ejecta layer is fully consistent with the 14.808 ± 0.038 Ma age of the Ries impact structure^17^. All results taken together, i.e., our palynological and paleontological investigations that reveal that mudstone and limestone clasts recovered from the Blockhorizont have ages that are consistent with equivalent lithologies in the Ries target stratigraphy, as well as the reported geochemical analysis, are consistent with clasts being derived from the Ries area, and, along with the age estimate of the horizon, support the hypothesis that the Blockhorizont is in fact indeed derived from the Ries impact. This means that the Blockhorizont constitutes the most distal horizon of distal Ries ejecta (not including the moldavite tektites). On this basis, the inventory of outcrops where distal Ries ejecta has been described from southern Germany can be confidently extended to eastern Switzerland (Fig. 1; e.g., Refs.^13,24^).

In principle, both quartz-bearing clasts and individual quartz grains could be derived from the sedimentary target rocks at the Ries. Sandstones of Triassic and Jurassic age are part of the target rocks, as well as surficial unconsolidated sands^38^; however, the clasts and the individual grains are very angular, and there is no evidence of rounding of grains comprising the fragments. Only one possible exception would be the fragment in Supplementary Fig. S2a,b, that could be sedimentary in origin, but this is not conclusive. Mineral assemblages of the lithic clasts (quartz, muscovite, biotite, and altered unidentified minerals) agree with a basement origin (such as granites and gneisses; Ref.^13^ and references therein). Our observations are thus consistent with the investigated quartz grains, as well as the majority of the investigated quartz-bearing fragments, being derived from the crystalline rocks located underneath the sedimentary cover, that were shocked and ejected during the Ries impact event.

### Emplacement of impact ejecta from the Ries at Bernhardzell

Understanding the formation and mode of emplacement of impact ejecta is fundamental in the evaluation of effects of an impact event on the biosphere^48^. Impact-generated dust and gas injection into the atmosphere was one of the driving forces of the mass extinction at the K–Pg boundary, leading to changes in solar irradiation reaching Earth’s surface which in combination with other (modelled) factors such as e.g., darkness, fireball effects, and increased thermal radiation created stressful conditions for biota, e.g., Ref.^49^. Smaller impact events may cause regional perturbation of the ecosystem, very much influenced by the ejecta distribution^48^.

Due to the very limited and poor preservation of terrestrial impact ejecta, but also because of the challenging identification of ejecta deposits in the geological record and also difficulties in tying layers to specific impact structures, the process of impact ejecta emplacement on Earth is relatively poorly understood. Analogies to other cratered bodies in the Solar System are not straight-forward, e.g., due to the differences in gravity, volatile content, and cohesiveness of target bedrock, e.g., Ref.^50^. Many Martian impact craters have, however, been discussed as being Earth-like in terms of ejecta deposition, e.g., Refs.^2,50^ (see also discussion in Ref.^51^).

The Blockhorizont contains material of different size ranges, stratigraphic position in the pre-impact setting, and with distinctly different mechanical properties (e.g., mudstone and lithic clasts derived from crystalline basement). We observe Upper Jurassic limestone blocks, up to 40-cm in size, with well-developed shatter cones, together with angular fragments of soft mudstone (of probable Miocene age), angular fragments of crystalline bedrock (up to 1 mm in size), and individual shocked quartz grains (tens to hundreds of micrometers in size) likely derived from the basement. In that sense, the Blockhorizont displays similar characteristics to ejecta from the Acraman impact structure (Australia), which also includes small fragments of individual mineral grains and larger fragments, up to 30 cm in size, that display shatter cones^51,52^, at a distance of 250–300 km from the structure. The Acraman ejecta horizon consists of two distinct grain size populations: poorly sorted breccia and larger clasts, and graded sand. It was suggested by Ref.^51^ that the material was deposited via different mechanisms during the impact event based on these two grain size populations, the breccia by ejecta-flow and/or fireball transport, and the graded sand by impact-induced air-blast transport. The Blockhorizont differs from the Acraman ejecta in that it is not composed of separate units, i.e., the quartz grains are found in the same interval as the limestone and mudstone fragments. The Acraman units are even separated by a sandy mud unit that was interpreted by Ref.^51^ as a short time gap between the arrivals of the different materials. However, the Acraman ejecta horizon occurs within deep-water shales, and the material was thus deposited in a vastly different setting than the ejecta that now forms the Blockhorizont, and the effects of gravitational settling through the water column will induce differences that make the two ejecta layers difficult to compare. In fact, Chicxulub distal ejecta particles, even if they were deposited via both ballistic and non-ballistic mechanisms, were deposited at roughly the same time (~ within weeks; e.g., Ref.^53^). During that impact event, numerical models demonstrate that distinct transport mechanisms for the different ejecta components are needed in order to match observational data and, specifically, distal transportation of single shocked quartz grains is proposed to be non-ballistic^53^. Distal impact ejecta emplacement for smaller impacts is a similarly complex process, despite the differences in magnitude, as discussed in Ref.^14^. Other works using numerical models and laboratory experiments to improve understanding of impact ejecta formation include e.g., Refs.^54,55^, and references therein.

In several models, the large limestone fragments in Ries ejecta are transported ballistically as early ejecta^14,47,54^. In the model by Ref.^14^, the early ejecta particles originate from the uppermost 50 m of the target sequence, and they experienced relatively low shock pressures (2–6 GPa). This could explain why the analyzed mudstone fragments are inconsistent with a Keuper origin, since Keuper mudstone belongs to a deeper portion of the target rocks (Supplementary Fig. S1). Low shock pressures recorded by these fragments also fit our observations. However, the model by Ref.^14^ does not reproduce the deposition of large fragments to the southwest of the Ries. It also shows that crystalline fragments which experienced shock pressures corresponding to those recorded by quartz grains in the Blockhorizont are not transported ballistically to such great distance. This indicates that the shock-metamorphosed grains of the current study were transported non-ballistically, or that our observations are not predicted by the model that was presented by Ref.^14^. Since models are simplified and many parameters (e.g., angle of impact and projectile speed) have bearing on aspects that are important to this work, e.g., how far ejecta is transported and the depth of excavation, we note that our observations can be used to improve models of ejecta emplacement, e.g., in testing whether the shocked quartz grains described in the present study were transported ballistically or non-ballistically. It is stated in Ref.^14^ that ballistic sedimentation of crystalline material preferentially occurs near the crater (i.e., closer than 40 km). Material comprising the distal ejecta of the Chicxulub impact was recently proposed to be transported in a fast-moving dust cloud of material from the ejecta curtain (in combination with material distributed from the impact plume)^56^. In this model, shocked mineral grains traveling at velocities of a few km s^−1^ could reach distal sites. Combined with previous models of distal ejecta distribution from Chicxulub^53^ that predict delivery of shocked quartz non-ballistically, this leads us to conclude that the presence of shocked quartz grains in the Blockhorizont can be explained by non-ballistic ejecta transportation, either through a mechanism related to re-distribution from a hot, expanding atmosphere (“impact plume”), or as material incorporated in a dust cloud. This separates the mineral grains from the large fragments that follow ballistic ejecta trajectories (see Ref.^53^). In Chicxulub distal ejecta, spherules are more or less abundant^57^, and these are distributed from the vapor plume^58^. In the Blockhorizont, no definitive impact spherules were recovered by us in the investigated samples. However, this is not surprising as recent modelling^58^ suggests that the average size of spherules for an impact equivalent in size to the Ries-forming event would be on the order of ~ 10 μm. We have to stress that such tiny spherules may be present in our samples but were overlooked as this size fraction was not investigated.

If non-ballistic deposition is the mechanism responsible for transporting these grains, possible effects that this impact may have had on the regional biosphere should not be neglected since material lifted and distributed by the impact plume (see e.g., Ref.^48^) or representing the fast-moving dense dust cloud^56^ formed by the Ries impact occurs in localities up to ~ 200 km away from the impact point. Although the most famous example of impact-induced environmental havoc is the far greater (in terms of energy release) Chicxulub/K–Pg event, the regional effects of smaller impacts was demonstrated, see e.g., Ref.^48^. In the case of the Ries ejecta, we have seen a vast difference in abundance pattern of clastic particles between the Blockhorizont and samples adjacent to the horizon. If we make the conservative assumption that the Blockhorizont is 5 cm thick, has a density of 2 g/cm^3^, and consists of ~ 2% impact ejecta (dominated by limestone; Table 1), ejecta amounts to ~ 2 kg/m^2^, indicating that the deposition of this layer must have been a significant disturbance in the area.

Because the Ries is a so-called rampart crater, or double-layered ejecta structure^23,50^, with analogues primarily described on the planet Mars, the study of ejecta from the Ries is also particularly valuable in furthering our understanding of ejecta characteristics on Mars, taking into account the differences that are expected in ejection and deposition processes due to the distinct conditions prevailing on Mars.

## Materials and methods

Samples from the Blockhorizont were collected from an outcrop on a bank of the Sitter river (9° 20.3′ E, 47° 28.78′ N; Fig. 1), near the town of Bernhardzell, ca. 6 km north-northwest of St. Gallen in eastern Switzerland (Figs. 1, 2, 3). Samples from bentonites were taken at Tiefenbachtobel near Engelburg, ca. 3 km northwest of St. Gallen for sample NMBE 43558, and at Rengishalden, 2.5 km west southwest of Bischofszell for sample NMBE 36981 (the Bischofszell bentonite). For sand grain content analysis of marl samples from the Bernhardzell Blockhorizont sampling site described above, samples were dried and disaggregated until complete dispersion, and then sieved. All figures were prepared using Adobe Photoshop and Illustrator 2020 software.

### Shocked quartz grains

The material (loose sediment constituting the Blockhorizont) studied for possible microscopic shock metamorphic features in minerals was first dispersed in water and then sieved (0.063, 0.1, 0.3, 1, 5 mm) in order to remove the clay fraction and also the larger fraction, i.e., fragments larger than 5 mm in size. The remaining fraction of the sample was then leached in HCl and mounted in epoxy blocks from which thin sections were prepared. The four thin sections where then searched for shocked quartz grains under an optical microscope. All quartz grains displaying PDFs were further studied using a Leitz 5-axis U-stage mounted on an optical microscope. Orientations of optic axes (c-axes) and poles perpendicular to PDF planes in quartz grains were measured and indexed following the technique described in Ref.^31^, see also references therein. The orientation of the poles perpendicular to planes of PDF and the optic axis of each quartz grain was plotted by hand on a stereographic Wulff net and then indexed with Miller-Bravais indices (hkil), using a stereographic projection template, displaying the possible pole orientations of common PDF planes within a 5° envelope of measurement error^31^. In order to attain precise angular relations, all the polar angles between PDFs and c-axis were further checked using the computer program Stereo32 (developed by K. Röller and C. Trepmann at Ruhr-Universität, Bochum, Germany).

The presence of planar fractures (PFs) was also noted, but their orientation was only measured in ten quartz grains, in order to obtain a general impression of their orientation pattern. All percentage calculations presented here represent absolute frequencies. An estimate of the frequency of shocked quartz in the sample was obtained by point counting and comparing the number of quartz grains displaying and devoid of PDFs in the investigated thin sections.

The thin sections were also studied using a Hitachi S-3400N scanning electron microscope at the Department of Geology, Lund University.

In addition, all shocked quartz grains were subjected to a roundness analysis to assess how the grains were affected by abrasion, based on the Pettijohn scale (e.g., Ref.^43^) and divided into the following categories: very angular (1), angular (2), sub angular (3), sub rounded (4), rounded (5), and well rounded (6).

### Biostratigraphy

For biostratigraphic purposes, a sample (~ 20 g) of the red mudstone from the Blockhorizont was processed using standard palynological methods at the Palynological laboratory, GEUS, Copenhagen, Denmark. The cleaned and crushed sample was digested in HCl and HF acids to remove carbonate and silicate minerals. After heavy liquid separation, to remove heavy minerals, and subsequent mild oxidation with Schulze’s solution, the organic residue was sieved on an 11 µm mesh. Three strew slides were curated with glycerin gel and carefully scanned for palynomorphs. The sample contained only low amounts of organic material.

Limestone fragments from the horizon were analyzed for their nannofossil and macrofossil content for age determination.

### Geochemistry

Whole rock element analysis was carried out on one sample of the red mudstone (clast within the Blockhorizont), one sample of loose sediment constituting the Blockhorizont, and on eight samples of Keuper clay clasts from the proximal Ries ejecta (the Bunte Breccia from the Gundelsheim, Oettingen, and Unterwilflingen localities). The concentrations of major and trace elements were determined by AcmeLabs, Canada, using an ICP-ES/ICP-MS. For REE normalization we used the CI chondrite values from Ref.^59^. The mineralogy (bulk and clay minerals) of these samples was determined by X-ray diffraction at the Institute of Geological Sciences, University of Bern. Bulk samples were measured on un-oriented samples, and clay minerals were analyzed on oriented samples of the fraction < 2 micron, both air-dry and after saturation with ethylene glycol. Lithium fluoride was used as an internal standard for the bulk mineralogy.

In addition, three limestone clasts, two of which show shatter cones, and one shatter cone-bearing limestone sample from the Steinheim impact structure (Germany; the Ries and Steinheim impact structures are considered to have formed simultaneously; see e.g., Ref.^13^), were analyzed for their oxygen and carbon isotopic composition using a ThermoFisher GasBench II linked to a Delta^plus^XL mass spectrometer at the University of Innsbruck, Austria. The δ^13^C and δ^18^O values are reported relative to the Vienna Pee Dee Belemnite (VPDB) standard. See Ref.^60^ for details on the method.

### Geochronology

Zircon grains from samples 36981 and 43558 were picked in ethanol, placed on double-sided sticky tape, and mounted in epoxy. The mount was polished with a diamond suspension to expose grain interiors before cleaning and carbon coating. The grains were imaged in backscattered electron (BSE) and cathodoluminescence (CL) modes on an FEI Quanta FEG 650 scanning electron microscope (SEM) at the Swedish Museum of Natural History, Stockholm, Sweden. The carbon coat was removed with a brief (less than 30 s) polish with 1 µm diamond polishing paste and after cleaning the mount was gold coated for in situ U–Pb analysis.

The grains were analyzed for U–Pb isotopic composition and age by secondary ion mass spectrometry (SIMS) on the Cameca IMS1280 ion microprobe at the NordSIMS Laboratory, Swedish Museum of Natural History. The methodology was modified from that described by Refs.^61,62^, specifically by utilizing the Hyperion H201 RF plasma high-brightness oxygen source. This produced an analysis pit approximately 10 µm across. Fifteen analyses were performed on fifteen separate grains in each of the two samples, all in a single analytical session. The zircon standard 91500 isotope dilute-thermal ionization mass spectrometry [ID-TIMS] ^206^Pb/^238^U age = 1062.4 ± 0.8 Ma; all uncertainties reported at 2σ unless otherwise stated; Ref.^63^ was used as the calibration reference material and Penglai (with an age of 4.4 ± 0.1 Ma^62^) was analysed as a quality control material. Six analyses on Penglai gave a weighted average ^207^Pb-corrected age of 4.41 ± 0.16 Ma (mean square of weighted deviates [MSWD] = 1.6; probability = 0.17), in agreement with the preferred age of Ref.^64^. Full U–Pb data for the two samples and Penglai are available in Supplementary Table S6.

## Supplementary Information

Supplementary Information.

Supplementary Table S3.

Supplementary Table S6.
