# Supplementary material for: Shocked quartz in distal ejecta from the Ries impact event (Germany) found at ~ 180 km distance, near Bernhardzell, eastern Switzerland
Source: Sci Rep. 2021 Apr 2;11:7438. doi: 10.1038/s41598-021-86685-2 (PMC8018947; doi:10.1038/s41598-021-86685-2)
Supplement: Supplementary file 1 — Supplementary Information. [file 41598_2021_86685_MOESM1_ESM.docx]

**SUPPLEMENTARY INFORMATION**

Shocked quartz in distal ejecta from the Ries impact event (Germany) found at ~180 km distance, near Bernhardzell, eastern Switzerland

**Sanna Holm-Alwmark, Carl Alwmark, Ludovic Ferrière, Matthias M. M. Meier, Sofie Lindström, Gavin G. Kenny, Emma Sheldon, Günter Schweigert, Christoph Spötl, Martin J. Whitehouse, and Beda A. Hofmann**

**The PDF file contains the following items:**

**Supplementary Figure S1: Pre-impact stratigraphy of Ries**

**Supplementary Figure S2: Photomicrographs of clasts from the Blockhorizont.**

**Supplementary Table S1: Supplementary observations from optical analysis of quartz grains.**

**Supplementary Table S2: Detailed results from mineralogical investigation.**

**Supplementary Table S3, see separate Excel-file.**

**Supplementary Figure S3: C-O cross plot.**

**Supplementary Table S4: Results of C and O isotopic analysis.**

**Supplementary Table S5: Results of palynological investigation of red mudstone from the Blockhorizont.**

**Supplementary Plate 1: Results of palynological investigation of red mudstone from the Blockhorizont.**

**Supplementary Text and Figure S4: Photos of ammonite specimens from limestone blocks from the Blockhorizont.**

**Supplementary Figure S5: U-Pb Concordia diagrams.**

**Supplementary Text and Table S6: U-Pb data, see also separate Excel-file.**

**References**

**Supplementary Fig. S1: Pre-impact stratigraphy Ries**

Pre-impact stratigraphy of the Ries area. Stars mark approximate pre-impact locations of ejecta particles that we recovered in the Blockhorizont. 1: quartz grains and lithic clasts, 2: limestone fragments, 3: red clay fragments. Figure modified after von Engelhardt (1990).

**Supplementary Fig. S2: Photomicrographs of clasts from the Blockhorizont**

**
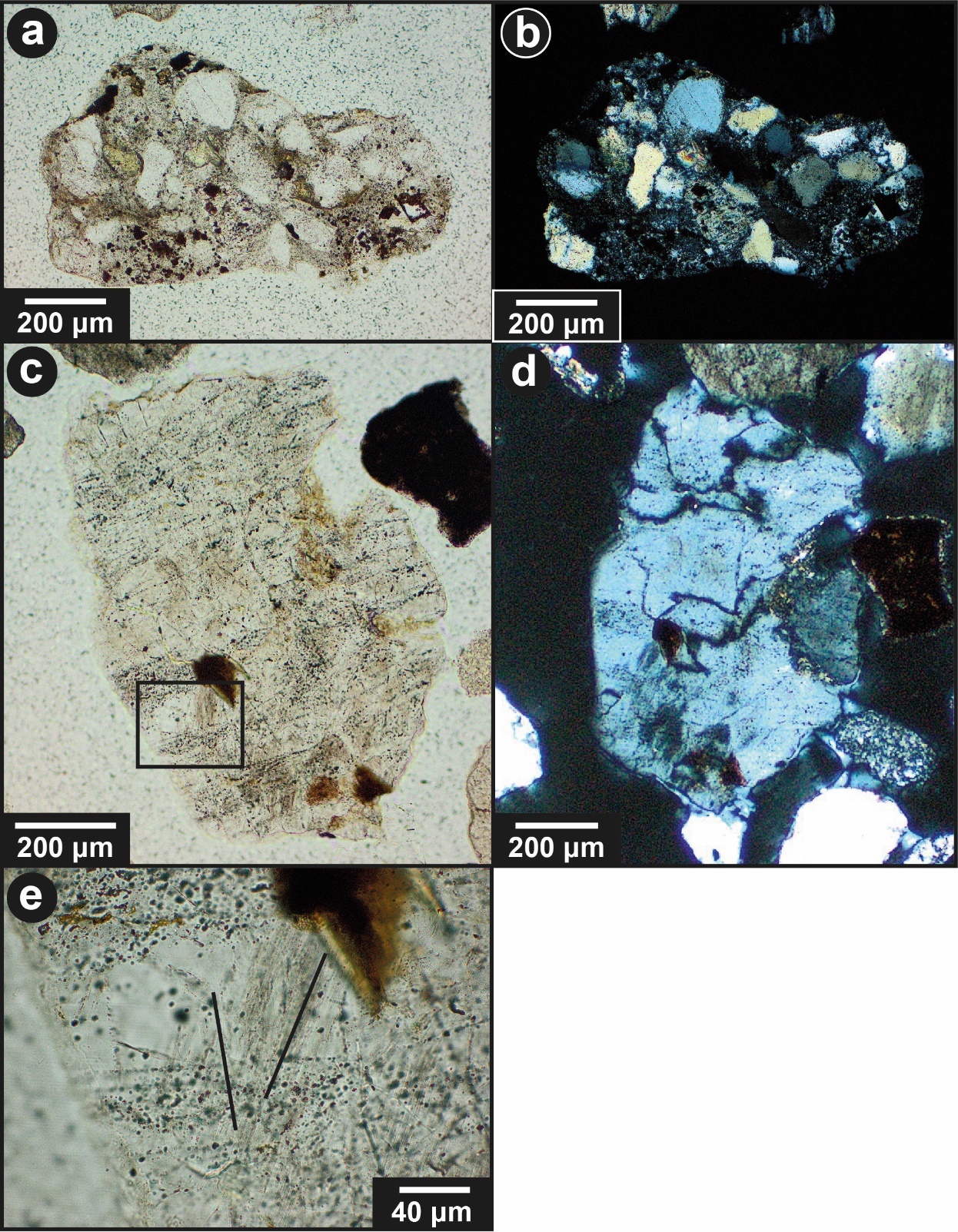
**

Supplementary Fig. S2. a) Rock fragment with quartz and altered phases (plane-polarized light; PPL). b) Fragment from a), in cross-polarized light (XPL). c) Rock fragment with quartz and dark phase (PPL). Box indicates area that is enlarged in e). d) Fragment from c), in XPL. e) PDFs (indicated by black lines) in quartz grains in rock fragment.

**Supplementary Table S1. Supplementary observations from optical analysis of quartz grains.**

| Supplementary Table S1. Summary of universal stage data for PDF set abundances and crystallographic orientations of these sets | | | | | | | | |  |  |  |  |
| --- | --- | --- | --- | --- | --- | --- | --- | --- | --- | --- | --- | --- |
| Sample | BZ |  |  |  |  |  |  |  |  |  |  |  |
| No. of investigated grains | 51 |  |  |  |  |  |  |  |  |  |  |  |
| No. of measured sets | 171 |  |  |  |  |  |  |  |  |  |  |  |
| No. of measured sets* | 158 |  |  |  |  |  |  |  |  |  |  |  |
| No. of PDF sets/grain (N) | 3.4 |  |  |  |  |  |  |  |  |  |  |  |
| No. of PDF sets/grain (N')* | 3.2 |  |  |  |  |  |  |  |  |  |  |  |
| Relative abundance of PDF sets/grain (%) | |  |  |  |  |  |  |  |  |  |  |  |
| 1 set | 6.0 |  |  |  |  |  |  |  |  |  |  |  |
| 2 sets | 26.0 |  |  |  |  |  |  |  |  |  |  |  |
| 3 sets | 24.0 |  |  |  |  |  |  |  |  |  |  |  |
| 4 sets | 22.0 |  |  |  |  |  |  |  |  |  |  |  |
| 5 sets | 12.0 |  |  |  |  |  |  |  |  |  |  |  |
| 6 sets | 6.0 |  |  |  |  |  |  |  |  |  |  |  |
| 7 sets | 4.0 |  |  |  |  |  |  |  |  |  |  |  |
| Total | 100 |  |  |  |  |  |  |  |  |  |  |  |
| Indexed PDF crystallographic orientations (absolute frequency percent^†^) | | | | |  |  |  |  |  |  |  |  |
| c (0001) | 1.8 |  |  |  |  |  |  |  |  |  |  |  |
| {10$\bar{\text{1}}$4}^§^ | 11.7 |  |  |  |  |  |  |  |  |  |  |  |
| ω {10$\bar{\text{1}}$3} | 55.0 |  |  |  |  |  |  |  |  |  |  |  |
| Π {10$\bar{\text{1}}$2} | 11.1 |  |  |  |  |  |  |  |  |  |  |  |
| r, z {10$\bar{\text{1}}$1} | 1.8 |  |  |  |  |  |  |  |  |  |  |  |
| m {10$\bar{\text{1}}$0} | n.d^#^. |  |  |  |  |  |  |  |  |  |  |  |
| ξ {11$\bar{\text{2}}$2} | 0.6 |  |  |  |  |  |  |  |  |  |  |  |
| s {11$\bar{\text{2}}$1} | 1.2 |  |  |  |  |  |  |  |  |  |  |  |
| ρ {21$\bar{\text{3}}$1} | 5.8 |  |  |  |  |  |  |  |  |  |  |  |
| x {51$\bar{\text{6}}$1} | 0.6 |  |  |  |  |  |  |  |  |  |  |  |
| a {11$\bar{\text{2}}$0} | n.d. |  |  |  |  |  |  |  |  |  |  |  |
| {22$\bar{\text{4}}$1} | 2.3 |  |  |  |  |  |  |  |  |  |  |  |
| {31$\bar{\text{4}}$1} | n.d. |  |  |  |  |  |  |  |  |  |  |  |
| t {40$\bar{\text{4}}$1} | 0.6 |  |  |  |  |  |  |  |  |  |  |  |
| k {51$\bar{\text{6}}$0} | n.d. |  |  |  |  |  |  |  |  |  |  |  |
| Unindexed | 7.7 |  |  |  |  |  |  |  |  |  |  |  |
| Total | 100 |  |  |  |  |  |  |  |  |  |  |  |
| *Excluding unindexed sets. |  |  |  |  |  |  |  |  |  |  |  |  |
| ^†^See text for description of method. | | | | |  |  |  |  |  |  |  |  |
| ^§^PDFs with measured orientations that plot in the overlapping zone between {10$\bar{\text{1}}$4} and {10$\bar{\text{1}}$3} were treated as {10$\bar{\text{1}}$3}. See text and Ferrière et al. (2009) for details. | | | | | | | | | | | |  |
| ^#^n.d. = none detected |  |  |  |  |  |  |  |  |  |  |  |  |

The majority of the PDF sets (55% of the total) are oriented parallel to the {10$\bar{\text{1}}$3} orientation. The {10$\bar{\text{1}}$4} and {10$\bar{\text{1}}$2} orientations represent 12% and 11% of the total population of the indexed PDFs, respectively. In addition, planes parallel to the (0001), {11$\bar{\text{2}}$2}, {10$\bar{\text{1}}$1}, {11$\bar{\text{2}}$1}, {21$\bar{\text{3}}$1}, {22$\bar{\text{4}}$1}, {40$\bar{\text{4}}$1}, and {51$\bar{\text{6}}$1} orientations occur in minor amounts. Low-angle PDFs (e.g., with {10$\bar{\text{1}}$3}- and {10$\bar{\text{1}}$4}-orientations) generally cross entire grains whereas higher-angle PDFs (e.g., parallel to the {11$\bar{\text{2}}$1}-, {21$\bar{\text{3}}$1}-, and {22$\bar{\text{4}}$1}-orientations) are only visible in a limited area of the host grain. Note that 6% of grains contain only one set of PDFs. PDFs in these grains have {10$\bar{\text{1}}$3}, {10$\bar{\text{1}}$4}, and {10$\bar{\text{1}}$2}-orientations, which means that strictly speaking they cannot be indexed. For crystallographic reasons, establishing the c-axis and only one rhombohedral set of PDFs in quartz does not allow indexing of that plane (because exact a-axis orientation/s is unknown). However, when investigating samples that are known to contain shocked quartz (i.e., quartz grains with PDFs), the standard procedure among researchers has been to allow indexing of also these sets, despite issues raised above (e.g., Ferrière et al. (2009), Holm-Alwmark et al. (2018) and references therein).

**Supplementary Table S2. DETAILED RESULTS FROM MINERALOGICAL INVESTIGATION.**

| SUPPLEMENTARY TABLE S2. MINERALOGY OF BLOCKHORIZONT IMPACT HORIZON AND RIES EJECTA FRAGMENTS | | | | | | | | | | | |
| --- | --- | --- | --- | --- | --- | --- | --- | --- | --- | --- | --- |
| Sample | Quartz* | Calcite | Dolomite | Orthoclase | Hematite | Goethite | Clay minerals^†, §^ | Illite | Smectite | Kaolinite | Chlorite |
| BHI Bernhardzell impactite | 14 | 16 | 21 | 0 | 0 | 0 | 49 | 41 | 42 | 14 | 3 |
| K9 Bernhardzell red mudstone clast | 10 | 0 | 0 | 0 | 5 | 0 | 85 | 33 | 63 | 4 | 0 |
| K1 Ries^#^ | 26 | 0 | 0 | 23 | 2 | 0 | 49 | 42 | 0 | 58 | 0 |
| K2 Ries | 46 | 0 | 0 | 1 | 3 | 0 | 50 | 13 | 76 | 12 | 0 |
| K3 Ries | 33 | 0 | 0 | 9 | 1 | 0 | 57 | 28 | 6 | 66 | 0 |
| K4 Ries | 18 | 0 | 0 | 0 | 1 | 7 | 74 | 13 | 0 | 87 | 0 |
| K5 Ries | 26 | 0 | 0 | 8 | <1 | 0 | 66 | 29 | 14 | 11 | 46 |
| K6 Ries | 27 | 0 | 0 | 9 | <1 | 0 | 64 | 14 | 58 | 28 | 0 |
| K7 Ries | 24 | 0 | 0 | 6 | 1 | 0 | 69 | 14 | 58 | 28 | 0 |
| K8 Ries | 11 | 0 | 0 | 12 | 3 | 0 | 74 | 85 | 7 | 7 | 0 |
| *Quartz to goethite determined by quantitative X-ray diffraction of non-oriented bulk powders using LiF as internal standard, wt%. | | | | | | | |  |  |  |  |
| ^†^Clay minerals calculated as difference to 100%. | |  |  |  |  |  |  |  |  |  |  |
| ^§^Clay mineral relative percentages determined by quantitative X-ray diffraction of oriented samples of fraction <2 micron. | | | | | | |  |  |  |  |  |
| ^#^Samples K1-K8 are all clasts of red mudstone from Bunte Breccia from Ries. | | | |  |  |  |  |  |  |  |  |

**Supplementary Table S3. RESULTS FROM CHEMICAL ANALYSIS.**

See separate Excel-file.

**Supplementary Figure S3. C-O cross plot.**

Supplementary Fig. S3. C-O cross plot. BER=Bernhardzell limestone fragment samples. STE13_SC1=Steinheim limestone sample.

**Supplementary Table S4. RESULTS OF C AND O ISOTOPIC ANALYSIS.**

| SUPPLEMENTARY TABLE S4. RESULTS OF C AND O ISOTOPIC ANALYSIS OF LIMESTONE SAMPLES FROM THE BLOCKHORIZONT AND THE STEINHEIM IMPACT STRUCTURE. | | | | |
| --- | --- | --- | --- | --- |
| Sample | Location | Description | δ^13^C (‰, VPDB) | δ^18^O (‰, VPDB) |
| BER_37037 | Bernhardzell, Switzerland  Bernhardzell, Switzerland  Bernhardzell, Switzerland | Limestone clast with shatter cone | 2.27 | -2.30 |
|  |  |  | 2.45 | -2.16 |
|  |  |  | 2.36 | -2.28 |
| BER_38631 |  | Limestone clast with shatter cone | 1.95 | -2.35 |
|  |  |  | 1.99 | -2.24 |
|  |  |  | 1.98 | -2.31 |
| BER_42034 |  | Limestone clast | 2.36 | -2.11 |
|  |  |  | 2.31 | -2.26 |
|  |  |  | 2.31 | -2.21 |
| STE13_SC1 | Steinheim, Germany | Shatter cones in limestone | 2.20 | -3.89 |
|  |  |  | 2.14 | -3.90 |
|  |  |  | 2.15 | -3.94 |
|  |  |  |  |  |
|  |  |  |  |  |

**Supplementary Table S5. RESULTS OF PALYNOLOGICAL INVESTIGATION OF RED MUDSTONE FROM THE BLOCKHORIZONT.**

The preservation of the palynomorphs is generally poor, however, a minor number of palynomorphs were registered:

| Taxa | Number of specimens | Comments |
| --- | --- | --- |
| Ferns |  |  |
| *Deltoidospora toralis* | 3 | Spores of similar morphology, with Palaeozoic forms usually assigned to *Leiotriletes*, are known from the Late Palaeozoic and onwards. |
| *Deltoidospora* spp. | 2 | Spores of similar morphology, with Palaeozoic forms usually assigned to *Leiotriletes*, are known from the Late Palaeozoic and onwards. |
| *Gleicheniidites senonicus* | 1 | Spores of similar morphology are known from the Late Triassic and onwards, e.g., Traverse (2007). |
| *Punctatisporites* sp. | 1 | Spores of similar morphology are known from the Late Palaeozoic and onwards, see e.g., Balme (1995). |
| *Lophotriletes/Conbaculatisporites* sp. | 1 | Spores of similar morphology are known from the Late Palaeozoic and onwards, see e.g., Balme (1995). |
| Unidentified spore (small) | 1 |  |
| Gymnosperms |  |  |
| Cheirolepidiaceae |  |  |
| *Classopollis torosus* | 1 | Late Triassic – K/Pg (Paleogene; see e.g., Tosolini et al., 2015, and references therein) |
| Cupressaceae |  |  |
| *Taxodiumpollenites* *hiatus* | 1 | This type of pollen is known from the Early Cretaceous and onwards, see e.g., Traverse (2007). |
| Ginkgoales/Cycadales |  |  |
| *Monosulcites* spp. (small) | 3 | Monosulcate pollen are known from the Carboniferous and onwards, see e.g., Traverse (2007). |
| Pinaceae |  |  |
| *Pinuspollenites* sp. cf. *P. minimus* | 2 | Similar pollen are known from Late Triassic to recent, see e.g., Traverse (2007). |
| Caytoniales |  |  |
| *Vitreisporites pallidus* | 1 | Known from the Permian to the latest Cretaceous (Maastrichtian), see e.g., Balme (1995). |
| Unidentified bisaccate pollen | 3 |  |
| Unidentified pollen | 4 |  |
| Angiosperms |  |  |
| Tricolpate pollen (small) | 1 | The oldest confirmed dispersed angiosperm pollen are of Barremian age, see e.g., Traverse (2007). |
| Phytoplankton |  |  |
| Dinoflagellate cysts |  |  |
| Unidentified dinoflagellate fragments | 9 |  |
| cf. *Pseudokomewuia granulata* | 1 | *P. granulata* is a freshwater dinoflagellate cyst known from the Oligocene to Miocene (Batten et al., 1999). |
| cf. *Cleistosphaeridium placacanthum* | 1 | In NW Europe *C. placacanthum* has its last occurrence (LO) at the base of the *Hystrichosphaeropsis obscura* Zone, i.e., within the upper Tortonian, at c. 8.8 Ma (Dybkjær & Piasecki, 2010; Dybkjær et al., 2020). |
| Acritarchs and prasinophytes |  |  |
| *Leiosphaeridia* sp. (small) | 1 |  |
| *?Cymatiosphaera* sp. (small) | 1 |  |
| Unknown |  |  |
| Unidentified palynomorphs | 3 |  |
| Total sum: | 41 |  |

**Supplementary Plate 1.**

Photographs of selected palynomorphs present in the red mudstone. Scale bar in F is 20 µm and applies to all photographs.

A–B. Unidentified cavate dinofagellate cyst resembling members of the cornucavate freshwater dinoflagellate cyst *Pseudokomewuia granulata*, known from Oligocene to Miocene strata^67^. Bernhardzell, red claystone, slide 4, P54/2.

C–D. Unidentified dinoflagellate cyst, cf. members of *Gochteodinia* sp. or *Prolixosphaeridium* sp. Bernhardzell, red claystone, slide 6, N39/1.

E. Small unidentified dinoflagellate cyst. Bernhardzell, red claystone, slide 5, O45/3.

F–G. Unidentified dinoflagellate cyst. Bernhardzell, red claystone, slide 4, C17/2.

H. Unidentified small dinoflagellate cyst, cf. *Cleistosphaeridium placacanthum* from the Miocene*.* Bernhardzell, red claystone slide 6, D27/2.

I. Fragment of unidentified dinoflagellate cyst showing slender, solid processes with multifurcating tips. Bernhardzell, red claystone, slide 5, O51/3.

J. *Gleicheniidites senonicus*. Bernhardzell, red claystone, slide 4, Z18/2.

K. *Classopollis torosus.* Bernhardzell, red claystone, slide 6, N36/3.

L. *Vitreisporites pallidus.* Bernhardzell, red claystone, slide 4, K35/2.

M. *Taxodiaceaepollenites hiatus*. Bernhardzell, red claystone, slide 6, Y44/1.

N. *Tricolpites* sp. Bernhardzell, red claystone, slide 5, K37/4.

O. Small granulate, ?triporate pollen. Bernhardzell, red claystone, slide 5, U30/3.

P. *Deltoidospora toralis.* Bernhardzell, red claystone, slide 5, V18/1.

Q. *Punctatisporites* sp., poorly preserved, trilete mark is visible in the lower part of the specimen. Bernhardzell, red claystone, slide 5, S31/4.

R. *Pinuspollenites minimus.* Bernhardzell, red claystone, slide 6, K30/4.

S. *Pinuspollenites minimus.* Bernhardzell, red claystone, slide 5, K28/4.

*
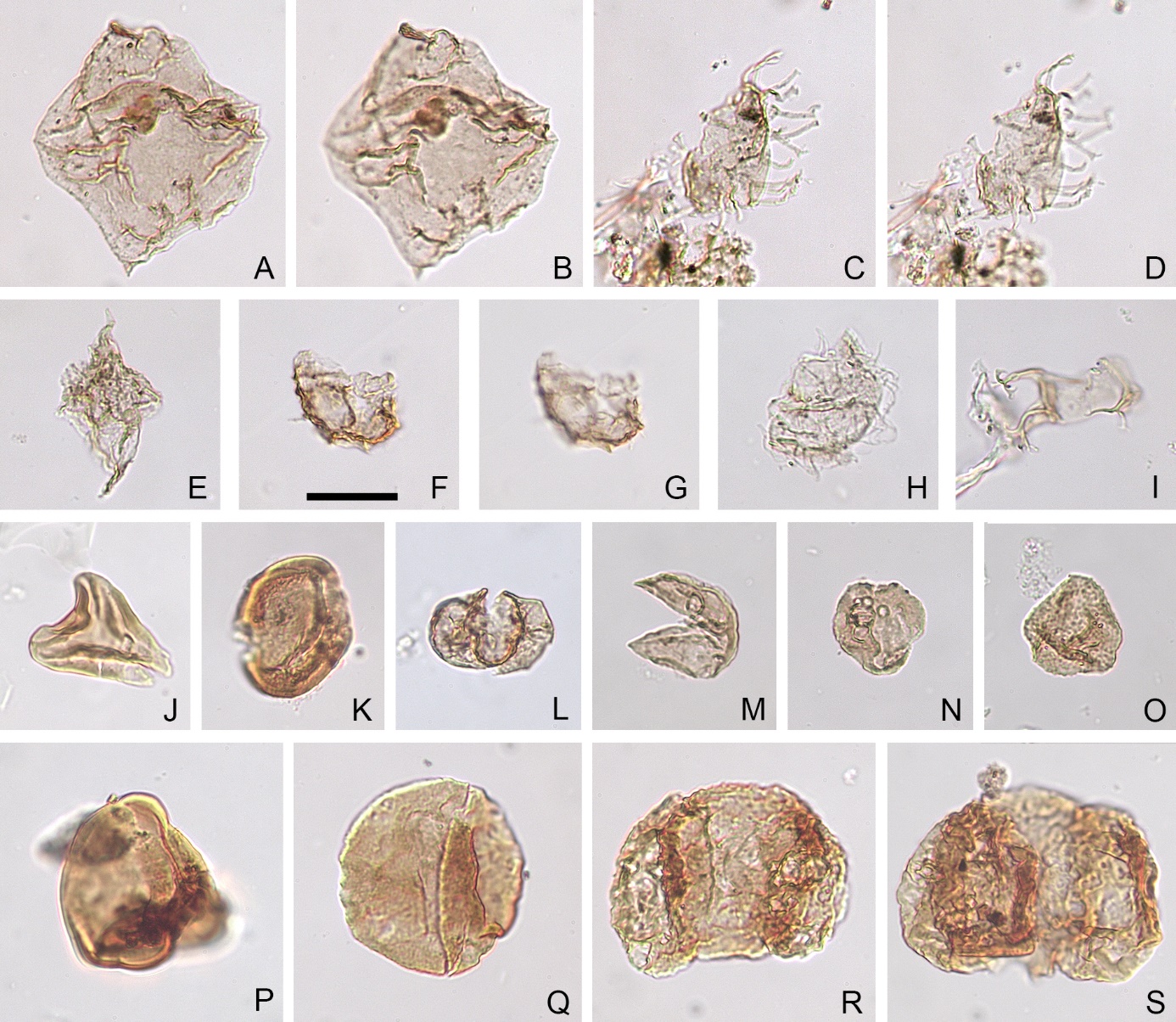
*

**Supplementary Text and Figure S4. PHOTOS OF AMMONITE SPECIMENS FROM LIMESTONE BLOCKS FROM THE BLOCKHORIZONT.**

Streblites levipictus is an oppeliid ammonite that occurs frequently in the Upper Jurassic Acanthicum Zone and lower part of the Pseudomutabilis Zone of Swabia and Franconia. Creniceras dentatum is a very characteristic microconchiate species, which represents the male of Streblites spp. Both taxa point to a Kimmeridgian age of the host rock.

Aspidoceratids occur from the Lower Kimmeridgian onwards. Since a closer generic or specific determination is impossible, the sole specimen only confirms a Late Jurassic age. The Taramelliceras sp. and the perisphinctid point to a Late Jurassic age as well, but do not allow a closer stratigraphic limitation due to their fragmentary preservation and multiple homoeomorphic forms present in the Upper Jurassic.


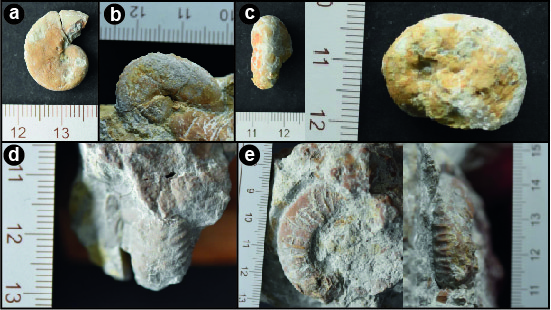


Supplementary Fig. S3. Ammonite specimens from limestone blocks, Bernhardzell: a) *Streblites levipictus* (det. G. Schweigert, 20.10.2016); b) *Creniceras dentatum* (det. R.A. Gygi, 1991); c) aspidoceratid ammonite (a-c Natural History Museum Bern, Inv. No. 42030); d) *Taramelliceras* sp. (det. R.A. Gygi, 1991; Natural History Museum Bern, Inv. No. 42031), e) perisphinctid ammonite (Natural History Museum Bern, Inv. No. 50064). Photographs taken by Ursula Menkveld-Gfeller, Natural History Museum Bern.

**Supplementary Figure S5. U-Pb Concordia diagram.**

Supplementary Fig. S5. Tera-Wasserburg concordia diagram showing zircon U–Pb data for bentonite samples 36981 and 43558. Data shown here are uncorrected for common Pb (Pb_c_) and plotted at 2σ uncertainty. Four other grains from sample 43558 were analyzed and gave Paleozoic and older ages that indicate they are detrital grains from strata adjacent to the bentonites - for clarity, only Miocene-aged analyses are plotted here.

**/**

**Supplementary Text and Table S6. RESULTS FROM U-Pb ANALYSIS.**

See also separate Excel file. Sample NMBE 36981 (the Bischofszell bentonite) gave a weighted mean ^207^Pb-corrected age of 14.53 ± 0.18 Ma (2σ, mean square of weighted deviates [MSWD] = 1.8, probability = 0.039, one of 15 analyses excluded). The single analysis excluded by the weighted mean function in Isoplot (Ludwig, 2012) is the youngest analysis, and may have given an erroneously young date as a result of post-deposition Pb-loss. The somewhat high MSWD (>1.5) indicates that there likely remains a small amount of geological scatter in the data, e.g., as a result of later Pb-loss giving dates that are slightly too young, and/or prolonged pre-eruption magma chamber residence times giving dates slightly older than the eruption age.

Sample NMBE 43558 (the Tiefenbachtobel bentonite) displays more spread in U-Pb dates. Four grains give much older dates of *ca.* 325, 465, 630, and 1575 Ma, respectively. The remaining 11 analyses spread between *ca.* 16 Ma and 14 Ma. We confirm that this horizon, located above the Blockhorizont at Tiefenbachtobel, is a bentonite, of Miocene age. This bentonite is much thinner, so it is not surprising that older, detrital grains from adjacent strata could have made their way into the sample. The eleven Miocene-aged grains give a weighted mean ^207^Pb-corrected age of 14.79 ± 0.34 Ma (2σ, MSWD = 5.2, probability = 0.000, none of 11 analyses excluded), indistinguishable from the age of sample NMBE 36981. The high MSWD and probability of 0.000 indicates significant geological scatter, again related to either Pb-loss giving erroneously young dates, prolonged residence times giving pre-eruption ages, or a combination of the two. It is not clear from the spread of dates for individual analyses (Fig. 7) which played a more significant role.

**References**

Balme, B. E. Fossil in situ spores and pollen grains: an annotated catalogue. *Rev. Palaeobot. Palynol.* **87**, 81–323 (1995).

Batten, D.J., Gray, J. & Harland, R. Palaeoenvironmental significance of a monospecific assemblage of dinoflagellate cysts from the Miocene Clarkia Beds, Idaho, USA. *Palaeogeogr. Palaeoclimatol. Palaeoecol.* **153**, 161–177 (1999).

Dybkjær, K. & Piasecki, S. Neogene dinocyst zonation for the eastern North Sea Basin, Denmark. *Rev. Palaeobot. Palynol.* **161**, 1–29 (2010).

Dybkjær, K. *et al.* A new stratigraphic framework for the Miocene – Lower Pliocene deposits offshore Scandinavia: A multiscale approach. *Geol. J.* **56**, 1699–1725 (2020).

von Engelhardt, W. Distribution, petrography and shock metamorphism of the ejecta of the Ries crater in Germany: a review. *Tectonophysics* **171**, 259–273 (1990).

Holm-Alwmark, S., Ferrière, L., Alwmark, C. & Poelchau, M. Estimating average shock pressures recorded by impactite samples based on universal stage investigations of planar deformation features in quartz – Sources of error and recommendations. *Meteorit. Planet. Sci*. **53**, 110–130 (2018).

Ludwig, K. R. User's Manual for Isoplot Version 3.75–4.15: a Geochronological Toolkit for Microsoft Excel. *Berkeley Geochronological Center Spec. Publ.* **5,** 75 p. (2012).

Stacey, J.S. & Kramers, J.D. Approximation of terrestrial lead isotope evolution by a two-stage model. *Earth Planet. Sci. Lett.* **26**, 207–221 (1975).

Tosolini, A.-M. P., McLoughlin, S., Wagstaff, B. E., Cantrill, D. J. & Gallagher, S. J. Cheirolepidiacean foliage and pollen from Cretaceous high-latitudes of southeastern Australia. *Gondwana Res*. **27**, 960–977 (2015).

Traverse, A. *Paleopalynology* xviii + 813 (Springer, 2007).
